# A novel system for evaluating drought–cold tolerance of grapevines using chlorophyll fluorescence

**DOI:** 10.1186/s12870-015-0459-8

**Published:** 2015-03-11

**Authors:** Lingye Su, Zhanwu Dai, Shaohua Li, Haiping Xin

**Affiliations:** Beijing Key Laboratory of Grape Sciences and Enology and CAS Key Laboratory of Plant Resources, Institute of Botany, Chinese Academy of Sciences, Beijing, 100093 China; University of Chinese Academy of Sciences, Beijing, 100049 China; INRA, Institut des Sciences de la Vigne et du Vin, UMR 1287 Ecophysiologie et Génomique Fonctionnelle de la Vigne (EGFV), 210 Chemin de Leysotte, 33882 Villenave d’ Ornon, France; Key Laboratory of Plant Germplasm Enhancement and Specialty Agriculture, Wuhan Botanical Garden, Chinese Academy of Sciences, Wuhan, 430074 China

**Keywords:** Drought–cold stress, Electrolyte leakage, *Fv/Fm*, Grapevine, LT50

## Abstract

**Background:**

Grape production in continental climatic regions suffers from the combination of drought and cold stresses during winter. Developing a reliable system to simulate combined drought–cold stress and to determine physiological responses and regulatory mechanisms is important. Evaluating tolerance to combined stress at germplasm level is crucial to select parents for breeding grapevines.

**Results:**

In the present study, two species, namely, *Vitis amurensis* and *V. vinifera* cv. ‘Muscat Hamburg’, were used to develop a reliable system for evaluating their tolerance to drought–cold stress. This system used tissue −cultured grapevine plants, 6% PEG solution, and gradient cooling mode to simulate drought–cold stress. *V. amurensis* had a significantly lower LT50 value (the temperature of 50% electrolyte leakage) than ‘Muscat Hamburg’ during simulated drought–cold stress. Thus, the former had higher tolerance than the latter to drought–cold stress based on electrolyte leakage (EL) measurements. Moreover, the chlorophyll fluorescence responses of *V. amurensis* and ‘Muscat Hamburg’ were also analyzed under drought–cold stress. The maximum photochemical quantum yield of PS II (*Fv/Fm*) exhibited a significant linear correlationship with EL. The relationship of EL with *Fv/Fm* in the other four genotypes of grapevines under drought–cold stress was also detected.

**Conclusions:**

A novel LT50 estimation model was established, and the LT50 values can be well calculated based on *Fv/Fm* in replacement of EL measurement. The *Fv/Fm*–based model exhibits good reliability for evaluating the tolerance of different grapevine genotypes to drought–cold stress.

**Electronic supplementary material:**

The online version of this article (doi:10.1186/s12870-015-0459-8) contains supplementary material, which is available to authorized users.

## Background

Abiotic stresses are major factors that affect the growth, development, and productivity of crops. Most studies have mainly focused on individual stresses, such as cold, drought, and high salinity [1-3]. However, different stresses might occur simultaneously in the field; thus, crops can suffer from the superimposition of these stresses [4,5]. Hence, cross−breeding or marker−assisted breeding, which targets single abiotic stress, might be insufficient for enhancing the performance of crops in the field. Therefore, the combination of different stresses should be considered in evaluating tolerance and stress−related molecular mechanism [4,6].

Summer drought with heat waves has been noticed in grape−producing regions [7-9]. The mechanisms of drought–heat effects have also been reported in different plants [6,10]. In addition to summer drought, grapevine routinely suffers from dry winter; during this season, regions such as North China with extremely continental climate experience a low temperature and air humidity with little snow [11,12]. Frozen water in the soil supporting the main roots results in limited water use in the soil by grapevine plants during winter, on the contrary, transpiration by woody tissues (cuticular transpiration and lenticular transpiration) from grapevine canes is relatively high due to low humidity. All *Vitis vinifera* cultivars can’t be survival under natural condition in the main Chinese grape−producing areas in North China. To have economy income, all grapevine canes should be buried during winter, even if the temperature is higher than −10°C. This process requires more labor, and thus, increases product cost. Generally, extremely low temperature could damage the bud and cane of grapevines [13]. Moreover, the combination of drought–cold stress in winter in North China might result in death of shoots, even death of young trees such as in apple trees which can be survival under individual cold stress [14]. Even a special term ‘choutiao’ in Chinese is given for the phenomenon concerning death of shoots or whole trees due to drought stress under cold winter and some special culture management were developed to overcome drought–cold stress in apple trees [14].

Various evaluation methods are available for quantifying the tolerances to individual drought or cold stress in the laboratory [15]. Measuring electrolyte leakage (EL) is one of the most frequently used methods to assess plant tolerance in response to drought and low temperature [16,17]. Abiotic stresses induce cell membrane injury, leading to intracellular ion efflux. EL measurement can reflect the change of ion exosmosis, and determine the cell damage level. Half−lethal temperature (LT50) is widely considered to represent the low−temperature tolerance in plants. The LT50 value can be generally calculated by EL measurement defined as the temperature at which EL decreases to 50% of that under optimal growth conditions [18]. However, this method is time consuming [19]. Moreover, severe stress (e.g., freezing environment) could seriously damage the membrane structure and cause secondary stress to the samples, thus affecting the accuracy of the method [20]. Few studies have focused on the combination of the two stresses. However, the damages induced by drought and cold have several common characteristics. Both stresses may cause cell dehydration and accumulation of reactive oxygen species, resulting in damaged membrane and photosynthesis system at cellular level [21,22]. Consequently, tolerance to combined stress could be quantified through methodologies similar to those for each individual stress.

The negative impacts on photosynthesis have been widely studied under abiotic stresses, and chlorophyll fluorescence measurement has been proven as an efficient and reproducible tool for evaluating plant susceptibility index to drought [23,24] or low temperature [20,25] stresses. This method reflects the susceptibility to the damages of the photo system II (PSII) in the photosynthesis electron transport chains [26]. As a nondestructive diagnostic tool, chlorophyll fluorescence method shows more benefits compared with EL measurement, especially the more rapid process induces less secondary stresses to the samples. Moreover, different parameters (e.g., *Fo*, *Fv/Fm*, and qP) can be measured [25,27].

In the present study, we mimicked a drought–cold stress condition by coupling polyethylene glycol (PEG)−induced water−deficit hydroponic culture system with cooling environment. Fluorescence parameters were determined to evaluate the tolerance of grapevine to combined drought−cold stress. We established a novel model to estimate LT50 values using *Fv/Fm* measurement based on the correlation between the EL and chlorophyll fluorescence parameters of the grape leaves exposed under combined drought–cold stress condition. This model simplifies the evaluation of the damages caused by drought–cold stress. The proposed model can be readily applied to determine the tolerance of the grape germplasm and cross–progeny individuals to breed drought–cold–tolerant grapevines.

## Results

### Individual drought and cold tolerance of V. amurensis and ‘Muscat Hamburg’

**Figure 1:**
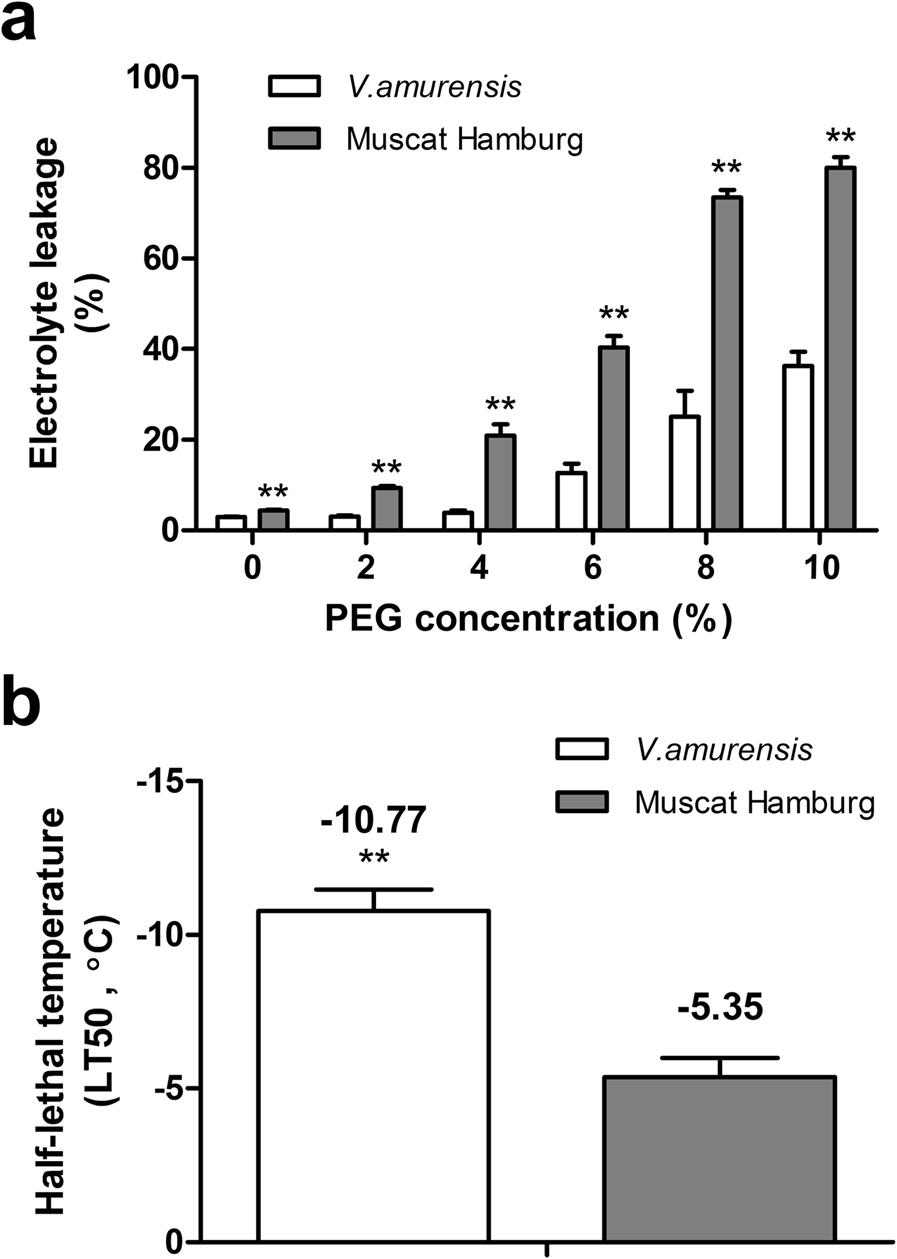
**Electrolyte leakages (a) under different concentrations of PEG for one day and electrolyte leakages based LT50 values (b) of grape leaves subjected to low temperature of** ***V. amurensis*** **and** ***‘*** **Muscat Hamburg’ plantlets.** The values represent the mean value±SE from five replicates and **indicates significant differences between *V. amurensis* and *‘*Muscat Hamburg’ at *P*<0.01 level (t test).

After exposure to PEG−simulated drought stress for 1 d, *V. amurensis* showed significantly lower EL than ‘Muscat Hamburg’ (*V. vinifera*) at all PEG levels (Figure 1a). *V. amurensis* showed a lower increase in EL than that of ‘Muscat Hamburg’ (12.2 vs 18.3 times) at 10% PEG compared with the controls. The EL difference between *V. amurensis* and ‘Muscat Hamburg’ increased as PEG concentration increased. Moreover, leaf relative water content (RWC) was lower in ‘Muscat Hamburg’ than that in *V. amurensis* under PEG stress, particularly at high PEG concentration (Additional file 1: Figure S1). RWC (75.8%, 68.0%, and 31.8%) was significantly lower in ‘Muscat Hamburg’ than that in *V. amurensis* under 6%, 8%, and 10% PEG treatments, respectively. The effect of the transpiration volume of the plantlets on the water potential of nutrient solution was also investigated. We filled the solution with distilled water to the initial volume every 12h after treatment. The two grape species exhibited significant phenotypic differences (Additional file 2: Figure S2).

To determine cold tolerance, we examined LT50 values calculated based on the measured ELs in both species. The LT50 values of *V. amurensis* and ‘Muscat Hamburg’ were −10.77 and −5.35°C, respectively; and they were significantly different in LT50 values between the previous two genotypes (Figure 1b). The different tolerances of *V. amurensis* and ‘Muscat Hamburg’ to the two individual stresses could be used as foundation for subsequent combined studies.

### Tolerances to drought–cold stress evaluated using EL–based LT50 value

To establish optimal conditions for combined stress, we performed a series of preliminary examinations for drought and low–temperature treating modes. A suitable PEG concentration should immediately trigger plant physiological responses and effectively discriminate drought tolerance among genotypes. However, the nutrient solution should remain unfrozen under the given freezing condition; freezing causes lower water potential [15] and therefore decreases the accuracy of the PEG concentration. According to these criteria and the results in Figure 1a, we selected 4%, 6%, and 8% as the candidate PEG concentrations. We then assessed the freezing pattern of the three PEG solutions at −6°C based on the pre−experiment, which showed that even *V. amurensis* exhibited severe water−soaking damage and EL almost reached the upper limit in all PEG concentrations at temperatures lower than −6°C. Moreover, 6% and 8% PEG remained unfrozen in the solution for 2 h (Additional file 3: Figure S3), whereas the solutions without PEG or with 4% PEG became frozen. Finally, 6% PEG, which induced moderate stress compared with 8% PEG, was selected for subsequent experiments.

**Figure 2:**
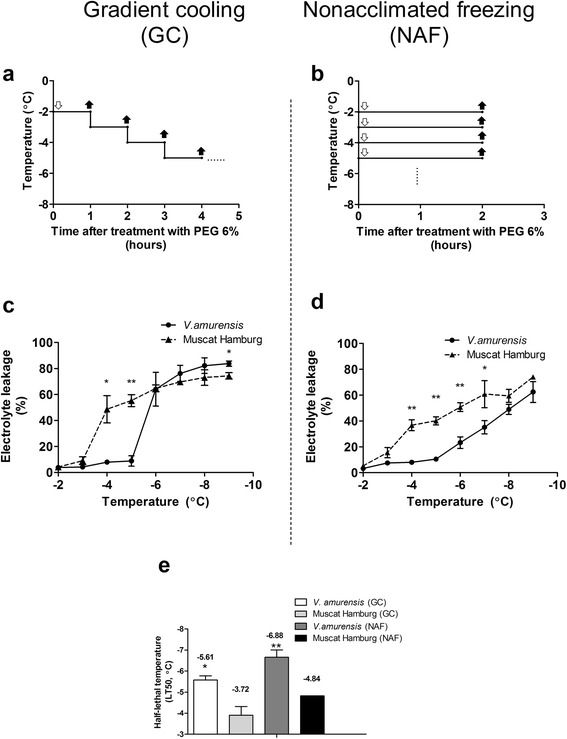
**Electrolyte leakages and LT50 values of** ***V. amurensis*** **and** ***‘*** **Muscat Hamburg’ plantlets under combined drought−cold systems. (a)** and **(b)** represent the pattern diagrams of different cooling modes. **(a)** Gradient cooling (GC) combined PEG 6% and continuous temperature decreased at a rate of 1°C/h from −2°C; **(b)** non−acclimated freezing (NAF) combined PEG 6% and directly frozen to each given temperature for 2 h. The feint arrows indicate the points when the plantlets began to subject cold stress, while the solid arrows represent the sample time at the end of each defined temperatures. **(c)** and **(d)** show electrolyte leakages under GC and NAF modes, respectively. **(e)** LT50 values of GC and NAF modes in *V. amurensis* and ‘Muscat Hamburg’. The values represent the mean value±SE from three to five replicates, * and ** indicate significant differences between *V. amurensis* and *‘*Muscat Hamburg’ at *P*<0.05 and *P*<0.01 level (t test), respectively.

EL was measured in both genotypes under 6% PEG coupled with simultaneous cooling treatment in both gradient cooling (hereafter referred to as ‘GC’, Figure 2a) and non−acclimated freezing (hereafter referred to as ‘NAF’, Figure 2b) modes. Low temperature significantly increased the EL values in both species; the increase in EL was higher in ‘Muscat Hamburg’ than that in *V. amurensis* (Figure 2c and d). EL was significantly different between the two genotypes from −4°C to −7°C under NAF mode, while the significant differences under GC mode were only observed at −4°C and −5°C. Under GC mode, the EL values in both genotypes slightly increased at initial degrees, whereas inflection point increased at high temperature in ‘Muscat Hamburg’ (−4°C, 5.38–fold increase) compared with that in *V. amurensis* (−6°C, 7.23–fold increase). Moreover, EL slowly increased under NAF mode compared with that under GC mode. LT50 values were calculated based on the EL data. As shown in Figure 2e, the LT50 values of *V. amurensis* and ‘Muscat Hamburg’ were −5.61±0.19°C and −3.72±0.42°C under GC mode and −6.88±0.34°C and −4.84±0.13°C under NAC mode, respectively.

### Chlorophyll fluorescence response

**Figure 3:**
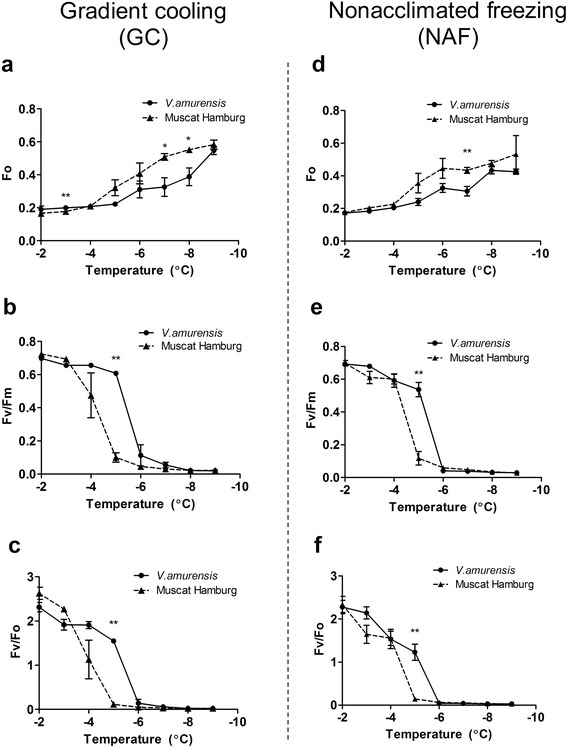
**Chlorophyll fluorescence response of** ***V. amurensis*** **and ‘Muscat Hamburg’ under two combined drought−cold stress modes. (a)**−**(c)** indicate the response of *Fo* **(a)**, *Fv/Fm* **(b)** and *Fv/Fo* **(c)** to gradient cooling (GC) mode, while **(d)**−**(f)** represent the response of *Fo* **(d)**, *Fv/Fm* **(e)** and *Fv/Fo* **(f)** to non−acclimated freezing (NAF) mode. The values were the mean value±SE of three replicates, and * and ** indicate significant differences between *V. amurensis* and *‘*Muscat Hamburg’ at *P*<0.05 and *P*<0.01 level (t test), respectively.

As shown in Figure 3, we examined three chlorophyll fluorescence parameters (*Fo*, *Fv/Fm*, and *Fv/Fo*) under drought–cold stress at the two cooling modes. *Fo* rapidly increased at temperatures lower than −4°C under both GC (Figure 3a) and NAF (Figure 3d) modes. Moreover, *Fo* was significantly higher in ‘Muscat Hamburg’ than that in *V. amurensis* at −6°C or/and −7°C. The *Fv/Fm* (Figure 3b and e) and *Fv/Fo* (Figure 3c and f) values decreased as temperature decreased under both cooling modes. In addition, a more rapid decrease of their values in ‘Muscat of Hamburg’ was observed than those in *V. amurensis* and significant difference was observed at −5°C at both cooling modes.

**Figure 4:**
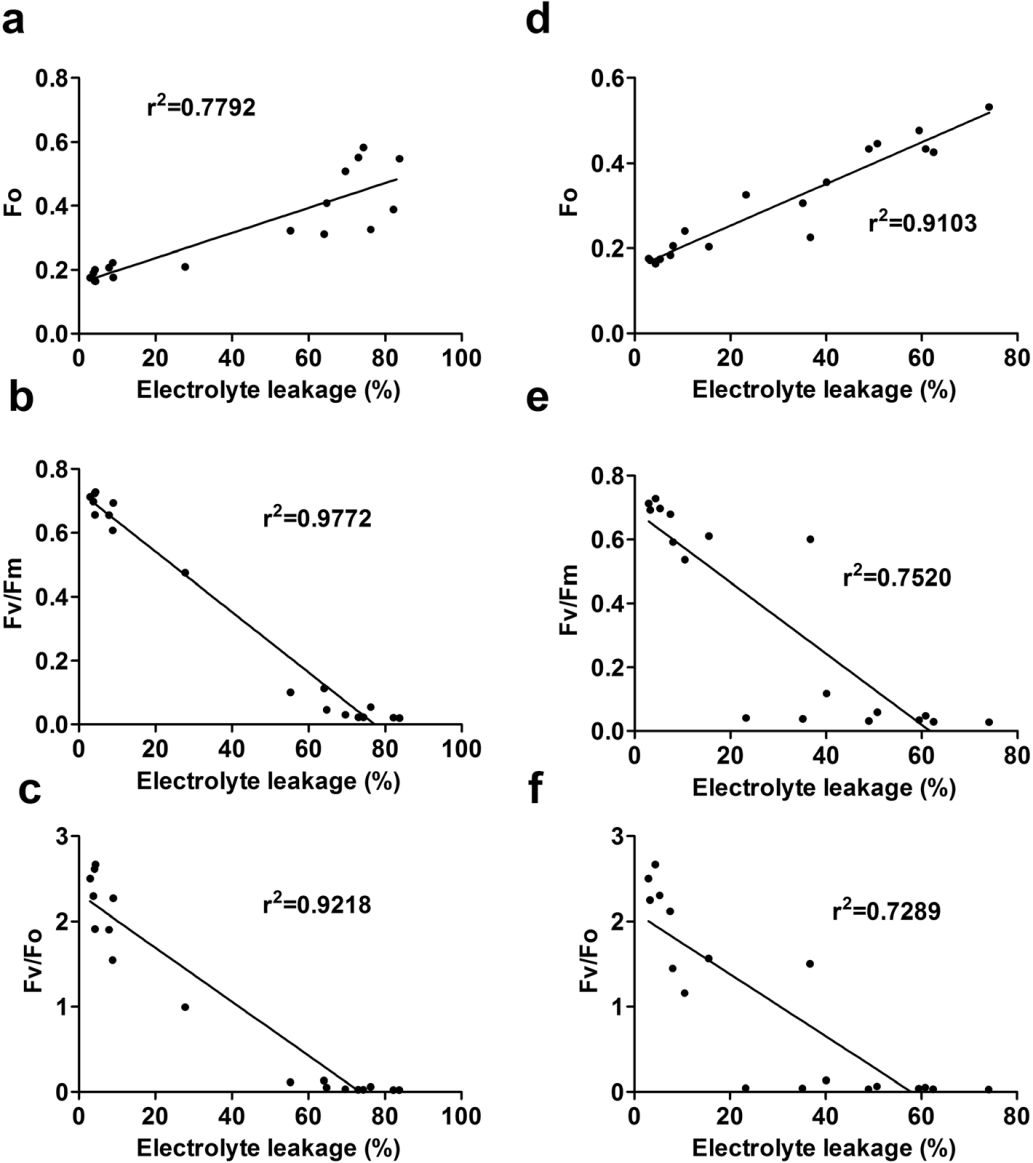
**Correlations between electrolyte leakage (EL) and three chlorophyll fluorescence parameters under two different drought−cold systems in** ***V. amurensis*** **and ‘Muscat Hamburg’. (a)**−**(c)**: Correlation between EL and *Fo* **(a)**, *Fv/Fm* **(b)** and *Fv/Fo* **(c)** under gradient cooling (GC) mode; **(d)**−**(f)**: Correlation between EL and *Fo* **(d)**, *Fv/Fm* **(e)** and *Fv/Fo* **(f)** under non−acclimated freezing (NAF) mode. Data were from those shown in Figures 2 and 3 as well as controls.

To establish an LT50 estimation model based on chlorophyll fluorescence responses, we should ensure a good correlation between EL and the candidate parameters. All the three chlorophyll fluorescence parameters were significantly correlated with EL under both cooling modes (Figure 4). Interestingly, the cooling modes affected the coefficient of correlation for different chlorophyll fluorescence−to−EL pairs. *Fv/Fm* and *Fv/Fo* showed higher correlations with EL under GC than those under NAF. The low correlation under NAF was mainly caused by the non−synchronous variation in the responses of chlorophyll fluorescence and EL to the decreasing temperatures. *Fv/Fm* and *Fv/Fo* reached their higher limits when EL was approximately 20%; thereafter, any further increase in EL (from 20% to 60%) was not accompanied by a proportional decrease in the two chlorophyll florescence parameters (Figure 3e and f). The *Fv/Fm* under GC showed the highest correlation with EL (*r*^2^=0.9772) among the three candidate parameters, and the two genotypes exhibited a unique regression line (Additional file 4: Table S1); thus, *Fv/Fm* was selected as the model for further analysis.

### LT50 estimation model under drought–cold stress based on chlorophyll fluorescence

**Figure 5:**
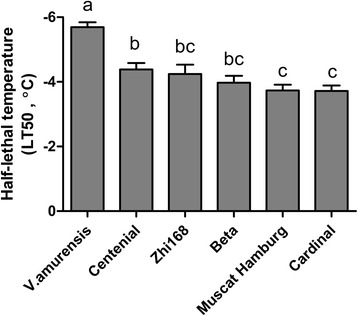
**The LT50 values obtained by EL under drought−cold stress (gradient cooling mode) in six different grape genotypes.** The bars were the±SE of three replicates and different letters indicate significant differences between the genotypes at *P*<0.05 (F test).

To confirm the reliability of our “PEG 6%+GC” system and the use of *Fv/Fm* as an alternative indicator of cold tolerance, we applied these parameters in the four other grape genotypes. Figure 5 shows the comparison between the LT50 values obtained from EL in the four newly investigated genotypes with those of the two genotypes used during system establishment under drought–cold stress. The lowest LT50 values were observed in *V. amurensis* at −5.61°C, whereas the highest in ‘Cardinal’ at −3.71°C.

**Figure 6:**
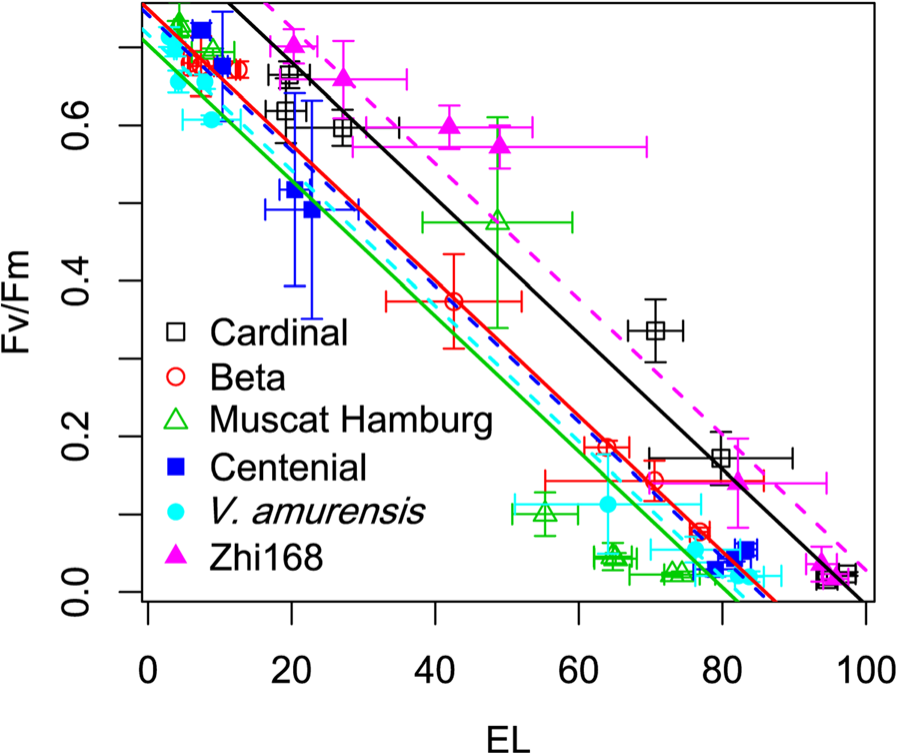
**Correlation between electrolyte leakage and** ***Fv/Fm*** **under drought−cold stress (gradient cooling mode) in six different grape genotypes (** ***V. amurensis*** **, ‘Muscat Hamburg’, ‘Centenial’, ‘Beta’, ‘Cardinal’ and ‘Zhi168’).** The values were the mean±SE of results from three replicates.

As shown in Figure 6, high correlations (*r*^2^>0.97, Additional file 5: Table S2) were observed between EL and *Fv/Fm* under GC mode for all the tested cultivars. In addition, all cultivars presented similar linear regression slope between *Fv/Fm* and EL; however, some differences were observed in their intercepts (Additional file 4: Table S1). This synchronization between the responses of *Fv/Fm* to EL under GC mode confirms the reliability of *Fv/Fm* as an effective indicator of cold tolerance. Therefore, we compared the LT50 estimated from *Fv/Fm* with the values estimated from classic EL values.

**Figure 7:**
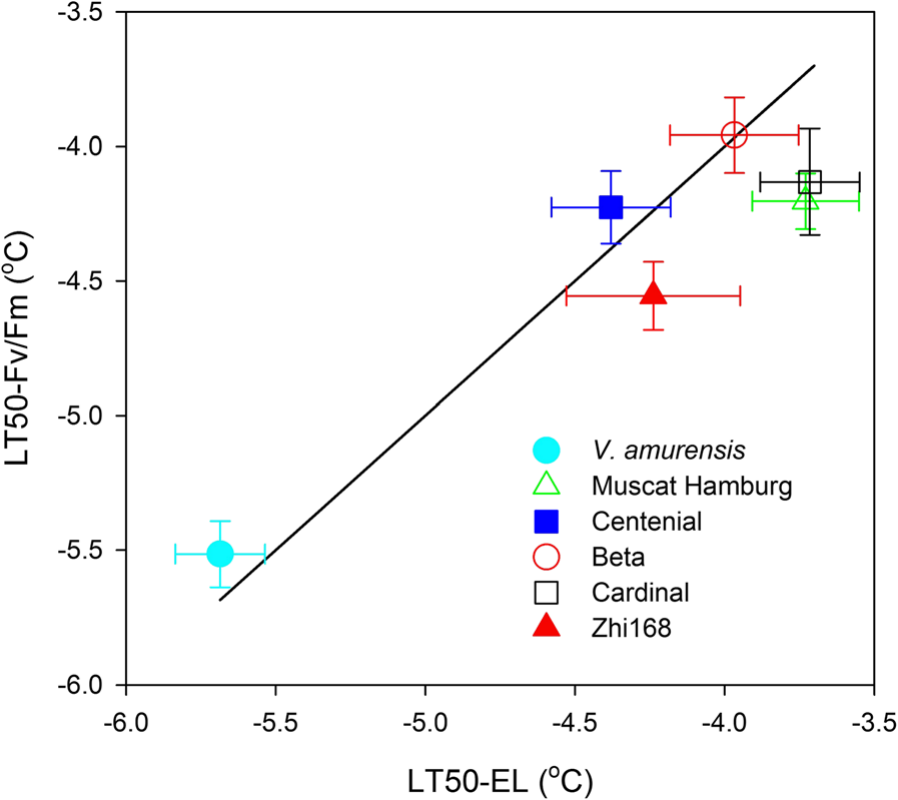
**Correlation between LT50 calculated based on EL and that calculated based on** ***Fv/Fm*** **in six different grape genotypes.** The values were the mean±SE of three replicates. The 1:1 line is presented.

Figure 7 and S4 demonstrate the comparison of the LT50 obtained from EL with those obtained from *Fv/Fm* under GC mode. A close correlation was observed between LT50–EL and LT50−*Fv/Fm* for all genotypes. The values of LT50–*Fv/Fm* were consistent with those of LT50–EL. A minor absolute difference of 0.3°C (RMSE), a low relative difference of 7.1% (RRMSE), and a very high agreement index of 93.4% were obtained. All these indexes indicate that LT50–*Fv/Fm* provides a reliable and precise representation of LT50–EL. Paired t tests have revealed that LT50−EL and LT50−*Fv/Fm* values were significantly different in the two genotypes (*V. amurensis* and ‘Muscat Hamburg’) under NAF, whereas no difference was observed under GC mode (Additional file 6: Table S3). This finding indicates that GC mode provided more consistent results between LT50−EL and LT50–*Fv/Fm*, and thus, more suitable for this system.

## Discussion

### Experimental system of combined stress

Mittler [4,28] emphasized that combined stress is not merely an addition of two individual stresses; the physiological and molecular mechanisms of combined stress should be studied and regarded as a novel stress. Some studies have elucidated the plant tolerance mechanisms to drought−heat [28], salinity−heat [29], drought−ozone [30], and drought–heat–virus [31]. However, the combination of drought and low–temperature stresses has been rarely reported except for the study on wheat [32]. This unique stress combination should be considered for actual fruit production. An accurate and simple method for evaluation is crucial for subsequent physiological and molecular research. The parents for breeding new cultivars with high resistance to the combined stress should be selected through stress evaluation at the germplasm level.

Establishing a suitable experimental platform for stress mimic is the prerequisite for evaluating drought–cold stress. In this study, the grape plant tissues cultured with 6% PEG solution under GC mode were subjected to a simulated drought–cold stress. PEG−induced hydroponic culture results in decreased water utilization by plants and is used for stable drought simulation because it is quantifiable and can be easily maintained. This culture condition is comparable with dry soil in winter; in which the frozen state causes unavailability of water in the upper soil layer, where most grapevine roots are distributed. In addition, *in vitro* grapevine hydroponic system exhibits rapid and easily reproducible abilities; this finding has also been observed on some other horticultural crops, such as apple [33], banana [34], sugar beet [35], and poplar [36]. By contrast to the classic method that uses detached leaves to evaluate cold tolerance [15], we used tissue−cultured grape plants to ensure consistency of plant material for investigating the whole−plant level.

### Tolerances to individual and combined stresses

*V. amurensis* is one of the most cold–tolerant species in the *Vitis* genus [37,38]; our present study confirmed this finding based on the lower LT50 value obtained (Figure 1b). *V. amurensis* also exhibits better drought tolerance caused by less membrane damage and water loss. To our knowledge, the comparison between the drought tolerance of *V. amurensis* with other grapes has been rarely reported [39], the result of which may broaden our understanding on the use of this genotype for evaluating combined stress. The combined stress was investigated under two different cooling modes with 6% PEG solution. EL assays showed that *V. amurensis* had significantly lower LT50 values than ‘Muscat Hamburg’ under both modes (Figure 2c and d). This finding indicates that *V. amurensis* had high tolerance to drought–cold stress. Remarkably, the LT50 values between *V. amurensis* and ‘Muscat Hamburg’ were similar under both cooling modes (about 2°C, Figure 2e). Thus, this evaluation method could significantly distinguish the tolerance of the two genotypes to the combined stress condition. The LT50 values of both genotypes under the combined stress were higher than the values under individual cold stress (Figure 1b). This result could be attributed to increased stress effect by drought stress. Adding PEG to induce drought stress might damage membrane stability, as indicated by the increasing LT50 values.

To determine the effects of the two different cooling modes combined with a fixed PEG concentration of 6%, we emphasized the relationship between EL and chlorophyll fluorescence measurements (*Fo*, *Fv/Fo*, and *Fv/Fm*) under both cooling modes. *Fv/Fo* and *Fv/Fm* exhibited significant higher correlations with EL under GC mode than those under NAF mode, indicating that GC mode was more suitable than NAF mode for drought−cold treatment. The changed trends of chlorophyll fluorescence data under two modes were similar (Figure 3), so the correlation differences possibly originated from the EL measurement results. This finding could be attributed to the insufficient time for increasing membrane damage within short−term freezing at defined temperature without pre−chilling accumulation; hence, EL did not exhibit a “steep−rise” at one inflection point under NAF mode, as opposed to that under GC mode. Under field conditions, the chilling temperatures in winter routinely and gradually decrease; therefore, GC mode could better mimic the natural environment than NAF mode. Indeed, an exponential decay regression might be better for correlating *Fv/Fm* and *Fv/Fo* to EL, particularly under the mode NAF (Figure 4e and f). However, the correlation *Fv/Fm* and EL under GC is clearly linear and an exponential decay regression may cause overfitting. Therefore, the linear regression was applied for all the correlations, which highlight the differences between the two cooling modes and provide support for better choice of GC mode.

In this study, we selected the chlorophyll fluorescence parameters from three designated indexes. Other parameters (e.g., coefficient of photochemical fluorescence quenching (qP), effective photochemical quantum yield of PS II, (ϕ_PSII_), and Stern-Volmer type non-photochemical fluorescence quenching (NPQ)) measured using chlorophyll fluorescence could also be used for model establishment [24]. However, compared with other parameters, the three candidate indexes could be easily obtained after dark adaptation without requiring actinic light adaption or far−red light illumination. Using these three parameters could provide a more convenient process that is not destructive to the leaf samples, and thus, is more advantageous for large−scale grapevine production and resistant breeding.

There are few points on all correlations of Figure 4 between 20% and 60% EL. This lack of evenly scattered points between 20% and 60% EL was a result of the sharp burst of cell damage occurred between −3 to −5°C (Additional file 7: Figure S4). Some suggestions to improve the system accuracy include to take an even smaller temperature decreasing gradient, e.g. changing from 1°C/h to 0.5°C/h between −3 to −6°C in GC mode. However, this will not only double the number of measurements but also challenge the accuracy of the cooling instruments. Consequently, the balance between the gain of accuracy and increase of manpower needs to be checked when applying the updated system to large scale drought−cold tolerance screening at population or germplasm level.

*Fv/Fm* is one of the most commonly used indexes for tolerance evaluation. However, using *Fv/Fm* mainly focuses on the tolerance to individual stress, such as pathogen [40], drought [41], freezing [20], and heat [42]. Some previous studies have reported that water deficit minimally affects *Fv/Fm* [23,43]; however, our preliminary experiments showed that *Fv/Fm*, as well as *Fv/Fo*, qP, and ϕ_PSII_, significantly decreased under individual drought stress (Additional file 8: Figure S5). The discrepancies in these studies could be attributed to the different growth conditions of the plant; the plants in the hydroponic system are more sensitive to drought than those grown in soil. The possible reason is the water potential may gradually decrease in soil dry, while the plants suffer from continuous given low water potential stress in the PEG−added hydroponic system during the whole treatment process, which leads to more rapid and severe damages in electron transport chains. Moreover, the findings suggest that the experimental system used in the present study could ensure that drought and cold stress, which were used as combined stress, individually affected the chlorophyll fluorescence results.

### LT50 estimation based on chlorophyll fluorescence parameter

The LT50 value is an easily comparable parameter for quantifying tolerance to drought and cold stresses [15]. However, classic LT50 calculation by measuring EL is time consuming and less accurate, and thus, unsuitable for large−scale screenings of drought−or cold−tolerant grapes. Hence, we established a suitable model to estimate the LT50 values without EL measurement. Determining chlorophyll fluorescence is a good alternative for EL measurement because of its non−invasiveness and rapidness, as well as its potential for estimating LT50 according to the high correlations between EL and given chlorophyll fluorescence parameters.

This study also reported a significant correlation between LT50–EL and LT50–*Fv/Fm* across different grape genotypes under simultaneous drought–cold stress; this correlation is beneficial for estimating LT50 without EL measurement. Moreover, investigating two markedly resistance−different genotypes, namely, *V. amurensis* and ‘Muscat Hamburg,’ and four genotypes increases the coverage in the spectrum of the natural drought–cold resistance of grapevine. The synchronization between the responses of *Fv/Fm* to EL under GC mode confirms the reliability of using *Fv/Fm* as an effective indicator of drought–cold resistance.

The significant correlation between LT50–EL and LT50–*Fv/Fm* has been observed under freezing condition in *Arabidopsis* (*Arabidopsis thaliana*) [20] and grape [19,44]. Interestingly, Ehlert *et al.* [20] emphasized that LT50–*Fv/Fm* is slightly lower than the LT50 value in *Arabidopsis* leaves. Jiang *et al.* [19] concluded that the *Fv/Fm* inflection point is higher than the LT50 of grape woody tissues. In the present study, LT50–EL and LT50–*Fv/Fm* are approximately equal, which may be due to the different cold sensitivities of plant tissues and the additive effect of drought and cold stresses.

The proposed evaluation system provides a more convenient and reliable tool for determining drought–cold resistance in the laboratory and for large–scale screening in the field. The system should be further improved before use for actual grapevine breeding.

## Conclusions

In the present study, we established and validated a novel experimental system for evaluating the resistance of grapevines against drought–cold stress. This system used tissue−cultured grape plants and 6% PEG solution under GC mode to simulate drought–cold stress. The resistance against drought–cold stress was evaluated in six different representative germplasms based on EL and chlorophyll fluorescence parameters, particularly *Fv/Fm*. A high correlation was observed between EL and *Fv/Fm*. Therefore, LT50 values can be well calculated based on *Fv/Fm* using the present system to evaluate the resistance of grapevine germplasms against drought–cold stress.

## Methods

### Plant material and culture conditions

Six genotypes of grape were pre−cultured on 1/2 B5 medium [45]. These genotypes included Chinese wild species *V. amurensis* (strongly tolerant to combined stress); three cultivars from *V. vinifera*, namely, ‘Muscat Hamburg’ (moderately tolerant to combined stress) [38], ‘Centenial’, and ‘Cardinal’; and two interspecific hybrids, namely, ‘Zhi168’ (*V. monticola* × *V. riparia*) and ‘Beta’ (*V. labrusca *× *V. riparia*). The plantlets with heights of 5–8 cm were transferred to 1/2 Hoagland nutrient solution in hydroponic boxes (37 cm × 8 cm × 5 cm) with continuous aeration. Culture conditions were 23±1°C and 60% relative humidity with 16−h light (120 μmolm^−2^s^−1^)/8−h dark photoperiod. After two weeks, the first three fully expanded leaves near the shoot apex were used for subsequent analysis.

### Evaluation of the individual resistance of V. amurensis and ‘Muscat Hamburg’ against drought and cold stresses

Individual resistance against drought and cold stresses was evaluated using the micropropagated plantlets of *V. amurensis* and ‘Muscat Hamburg’, which were acclimated in 1/2 Hoagland nutrient solution for two weeks. PEG−6000 was added into the solution to decrease water potential for mimicking drought stress. *V. amurensis* and ‘Muscat Hamburg’ were subjected to five different concentrations of PEG (2%, 4%, 6%, 8%, and 10%) for 1 d, whereas control plants were grown in a solution without PEG (CK). The third fully expanded leaf of each plantlet was sampled. The leaf samples were divided into two groups, which were subjected to EL (approximately 0.1 g) and RWC measurement. Moreover, chlorophyll fluorescence responses of the plantlets of both genotypes subjected to 6% PEG were evaluated using the third fully expanded leaf. All set of data had five replicates.

A classic method was used to assess the tolerance to a single cold stress [15]. Three leaf discs (6 mm in diameter) from the third fully expanded leaf were added into one tube containing 100 μL of distilled water. The tubes were transferred to a low−temperature incubator. After equilibrium at 0°C for 1 h, the temperature was decreased at a rate of 2°C/h from −2°C to −16°C. The samples were collected at defined temperatures to measure EL and LT50 values.

### Combined drought–cold treatments

To establish the drought–cold treatment system, we selected a suitable cooling mode combined with a fixed PEG concentration. To determine the PEG concentration, we added 100 mL of 1/2 Hoagland nutrient with different PEG concentrations (0%, 4%, 6%, and 8%). The solutions were distributed into flasks and placed in a specific freezing environment. The optimal PEG concentration was selected to effectively distinguish the drought tolerance among genotypes; PEG should be non−frozen at a given temperature. The plantlets in the selected PEG concentration (6%) were subjected to two different cooling modes for mimicking combined drought–cold treatments. The two cooling modes were as follows: (1) gradient cooling (‘GC’, Figure 2a): a given low temperature was maintained for 1 h and rapidly reduced by 1°C; the procedure was repeated from −2°C to −9°C (each temperature point had three replicates); and (2) non−acclimated freezing (‘NAF’, Figure 2b): direct freezing from normal growth temperature (23°C) to a given low temperature as GC mode and then maintained at the low temperature for 2 h (each temperature point had five replicates). The third fully expanded leaf attached to the plant was used for chlorophyll fluorescence measurement at defined temperature. The leaves were collected for EL and LT50 calculation. The chosen drought−cold treatment (PEG6%+GC mode) were also applied to four other genotypes (‘Centenial’ ‘Cardinal’, ‘Zhi168’ and ‘Beta’) as former two genotypes.

### Measurement of EL

EL was measured according to the method of Ma *et al.* [46] with some modifications. Briefly, the leaf samples exposed to drought, cold, and combined drought–cold stresses and their controls were collected and incubated in 6 mL of distilled water. After shaking at 0.5 *g* and 25°C for 3 h, initial conductivity (*C*1) was measured with a conductivity meter (FE30, METTLER TOLEDO, Switzerland). The samples were then autoclaved at 121°C for 20 min. After cooling to room temperature, the conductivity was re−measured as *C*2. EL was calculated using the equation EL (%)=*C*1/*C*2×100.

### Measurement of RWC

RWC was measured using the method of Sairam *et al.* [47] with minor modification. For fresh weight (FW), the collected leaves were immediately weighed. The leaves were added into 100 mL of distilled water and incubated at room temperature overnight. Subsequently, the leaves were removed from the water. The liquid on the surface of the leaves was immediately dried using a filter paper and then weighed as the turgid weight (TW). The samples were oven dried at 80°C for 10 h to determine the dry weight (DW). RWC was defined as RWC (%)=(FW−DW)/(TW−DW)×100.

### Measurement of chlorophyll fluorescence parameters

The third fully expanded leaf attached to the plant was subjected to a pulse−amplitude modulation fluorometer (PAM–2500, Walz, Germany) to determine chlorophyll fluorescence parameters. After 20–min dark adaptation, minimum fluorescence level (*Fo*) was determined with a low–intensity measuring light. Maximum fluorescence level (*Fm*) was measured after 0.5 s saturating pulse at 4,000 μmolm^−2^s^−1^. Steady–state fluorescence level (*Fs*) was obtained after 20–min actinic light (234 μmolm^−2^s^−1^) adaptation. Light–adapted maximum fluorescence level (*Fm*’) was measured with a second saturating pulse (0.5 s, 4,000 μmolm^−2^s^−1^). The actinic light was then closed, and light−adapted minimum fluorescence level (*Fo*’) was determined using a far–red light for 5s. Based on these parameters, we obtained four identification indexes: *Fv/Fm*=(*Fm*−*Fo*)/*Fm*, *Fv/Fo*=(*Fm*–*Fo*)/*Fo*, ϕ_PSII_=(*Fm*’−*Fs*)/*Fm*’, and qP=(*Fm*’−*Fs*)/(*Fm*’−*Fo*’). The four parameters, *Fv/Fm*, *Fv/Fo*, ϕ_PSII_, and qP, represent the maximum photochemical quantum yield of PS II, potential activity of PS II, effective photochemical quantum yield of PS II, and coefficient of photochemical fluorescence quenching, respectively [48-50].

### Model for estimation of LT50 based on leaf chlorophyll fluorescence response

Half−lethal temperature (LT50, the temperature at which the EL of leaf was reduced by 50%) was calculated by fitting the EL data to the Boltzmann 4 parameter model using R software [51].

$$ y={Y}_{\min }+\frac{Y_{\max }-{Y}_{\min }}{1+{e}^{\left(b\left(x-c\right)\right)}} $$

where *y* is the measured EL, *x* is the temperature, *Y*_min_ is the minimum value of EL, *Y*_max_ is the maximum value of EL, *b* is the slope at inflection temperature, and *c* is the inflection temperature, namely, LT50.

LT50 was calculated using the same equation by replacing EL with the selected chlorophyll fluorescence parameters.

To identify a reliable and non−infusive indicator of drought–cold tolerance, we used standardized major axis linear regressions. These equations are used for quantifying the relationships between the measured chlorophyll fluorescence parameters and EL, and for comparing their slopes among different genotypes [52].

The relationship between the obtained LT50 from the newly identified chlorophyll fluorescence parameter (LT50_new_) and that from the classic EL measurements (LT50_EL_) was quantified using the following criteria:

Root mean squared error: $RMSE=\sqrt{\frac{1}{N}{\displaystyle \sum_1^N{\left(LT{50}_{new}-LT{50}_{EL}\right)}^2}}$

Relative root mean squared error: $RRMSE=\frac{RMSE}{\overline{LT{50}_{EL}}}$

Agreement index: $index=1-\frac{{\displaystyle \sum_1^N{\left(LT{50}_{new}-LT{50}_{EL}\right)}^2}}{{\displaystyle \sum_1^N{\left(\vert LT{50}_{new}-\overline{LT{50}_{EL}}\kern0.5em \vert +\vert LT{50}_{EL}-\overline{LT{50}_{EL}}\kern0.5em \vert \right)}^2}}$where *N* is the number of genotypes used and $\overline{LT{50}_{EL}}$ is the average value of all LT50 obtained from EL measurements. Small RMSE and RRMSE values indicated better agreement between the two methods of LT50 estimation [53].

### Statistical analysis

Data are expressed as mean±SE. T−test was used to compare EL, LT50, RWC, and chlorophyll fluorescence parameters between *V. amurensis* and ‘Muscat Hamburg’. Paired t–test was used to compare LT50–EL and LT50–*Fv/Fm*, whereas the differences between the calculated and estimated LT50 among the six genotypes were analyzed through F–test by comparing the nested models [54].

## Additional files

Additional file 1: Figure S1. Effect of PEG concentration levels on relative water content in *V. amurensis* and ‘Muscat Hamburg’. The values were the mean value ± SE of results from five replicates. * and ** indicate significant differences between *V. amurensis* and *‘*Muscat Hamburg’ at *P*< 0.05 and *P*< 0.01 level (t test), respectively. Additional file 2: Figure S2. Comparison of plant growth conditions under PEG 6% treatment in V. amurensis and ‘Muscat Hamburg’. Distilled water was refilled into solution to keep the initial volume every 12 hours. Additional file 3: Figure S3. The ice frozen conditions of 1/2 Hoagland nutrient solution with different PEG concentrations at −6°C for 2 h. (a) control; (b) PEG 4%; (c) PEG 6%; (d) PEG 8%. Additional file 4: Table S1. Summary statistics of linear regressions between the electrolyte leakage and the chlorophyll fluorescence parameter *Fv/Fm* under gradient cooling mode in six different grape genotypes. Slopes and intercepts are estimated by standard major axis regressions for each genotype. Their 95% confidence intervals (CI) are also provided. Different letters indicate significant differences in the intercept or slope among the genotypes at *P*< 0.05. Additional file 5: Table S2. Correlation between electrolyte leakage (EL) and four chlorophyll fluorescence parameters (Fo, Fm, *Fv/Fm* and *Fv/Fo*) under gradient cooling mode in six different grape genotypes. Additional file 6: Table S3. Significant analysis of LT50−EL and LT50−*Fv/Fm* data in *V. amurensis* and ‘Muscat Hamburg’ under two different cooling modes. * indicates significant differences between LT50−EL and LT50−*Fv/Fm* at *P*<0.05 level (paired t test). Additional file 7: Figure S4. Responses of electrolyte leakage and *Fv/Fm* as a function of temperature in different grape genotypes. The open symbols are observed mean ± SE with three replicates and lines are fitted curves to the Boltzmann 4−parameter model. Filled symbols indicate where the LT50 were estimated and the corresponding genotype is indicated in each figure. Additional file 8: Figure S5. Comparison of *Fv/Fm* (a), *Fv/Fo* (b), qP (c) and ϕ_PSII_ (d) at different time after PEG 6% treatment in *V. amurensis* and ‘Muscat Hamburg’. The values were the mean value ± SE of results from five replicates. * and ** indicate significant differences between *V. amurensis* and *‘*Muscat Hamburg’ at *P*< 0.05 and *P*<0.01 level (t test), respectively.

## Abbreviations

EL: Electrolyte leakage
LT50: Half−lethal temperature
PEG: Polyethylene glycol
RWC: Relative water content
