# Supplementary figures and images for: A novel system for evaluating drought–cold tolerance of grapevines using chlorophyll fluorescence
Source: BMC Plant Biol. 2015 Mar 11;15:82. doi: 10.1186/s12870-015-0459-8 (PMC4367880; doi:10.1186/s12870-015-0459-8)

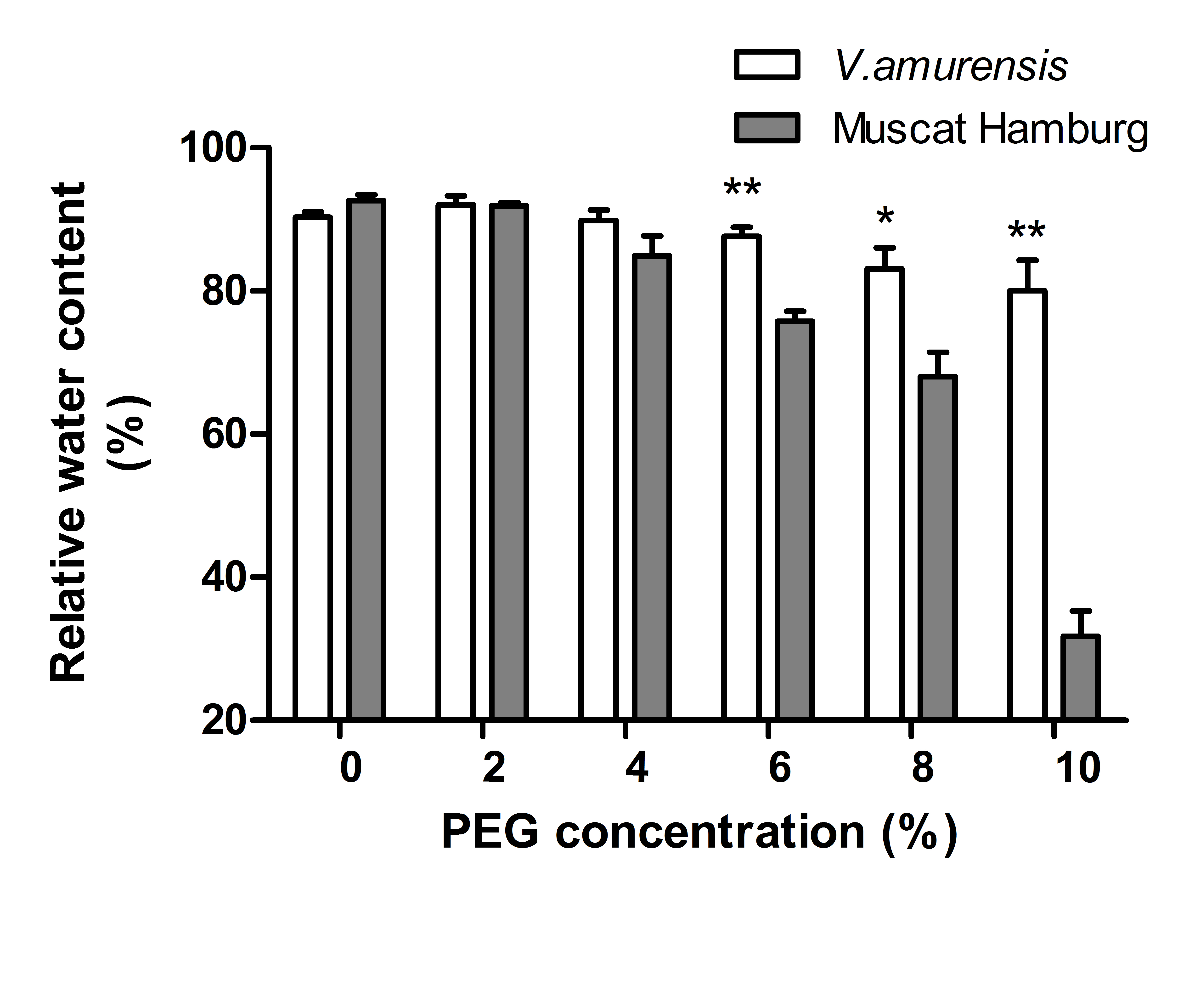

Supplement: Additional file 1: Figure S1. — Effect of PEG concentration levels on relative water content in V. amurensis and ‘Muscat Hamburg’. The values were the mean value ± SE of results from five replicates. * and ** indicate significant differences between V. amurensis and ‘Muscat Hamburg’ at P< 0.05 and P< 0.01 level (t test), respectively. [file 12870_2015_459_MOESM1_ESM.jpeg]

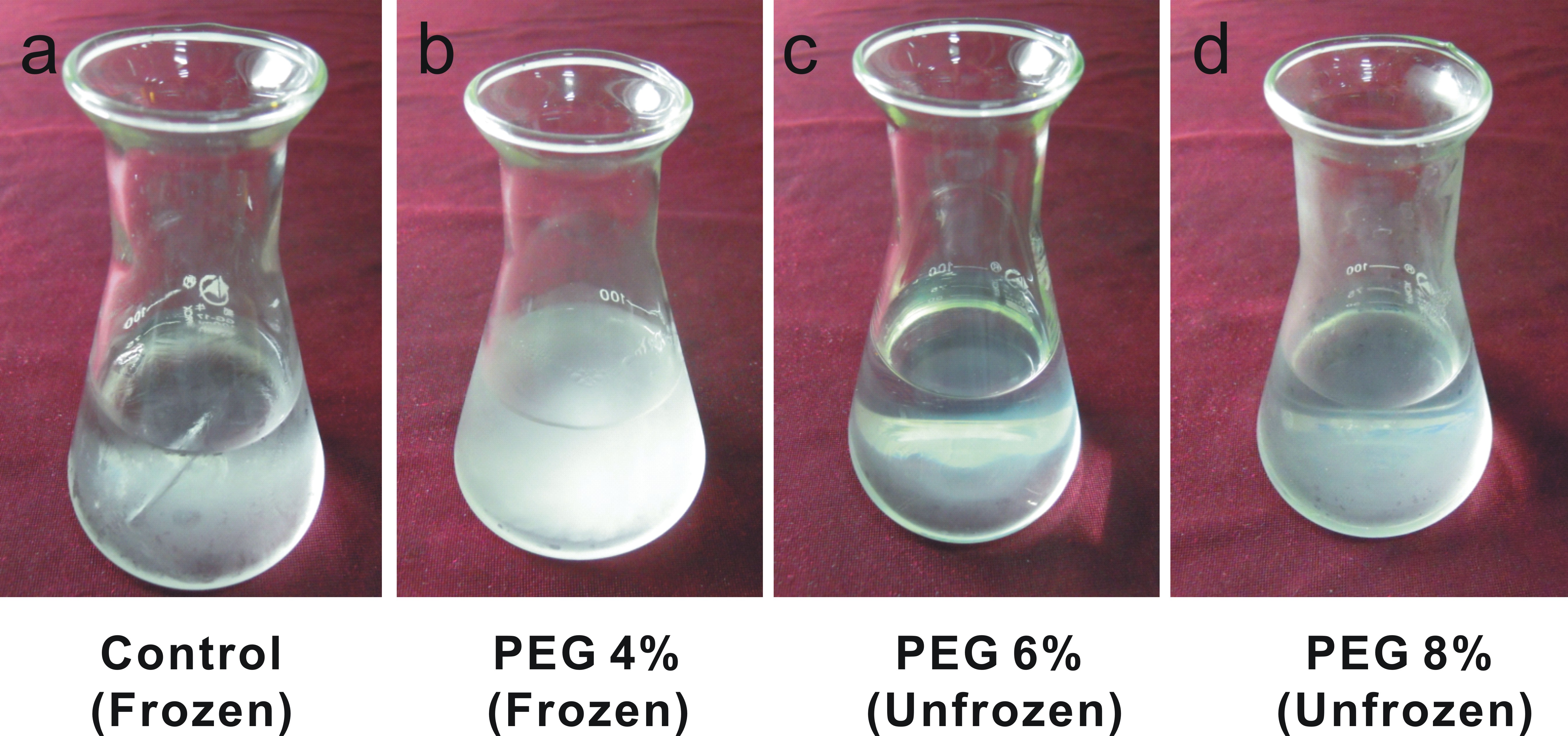

Supplement: Additional file 3: Figure S3. — The ice frozen conditions of 1/2 Hoagland nutrient solution with different PEG concentrations at −6°C for 2 h. (a) control; (b) PEG 4%; (c) PEG 6%; (d) PEG 8%. [file 12870_2015_459_MOESM3_ESM.jpeg]

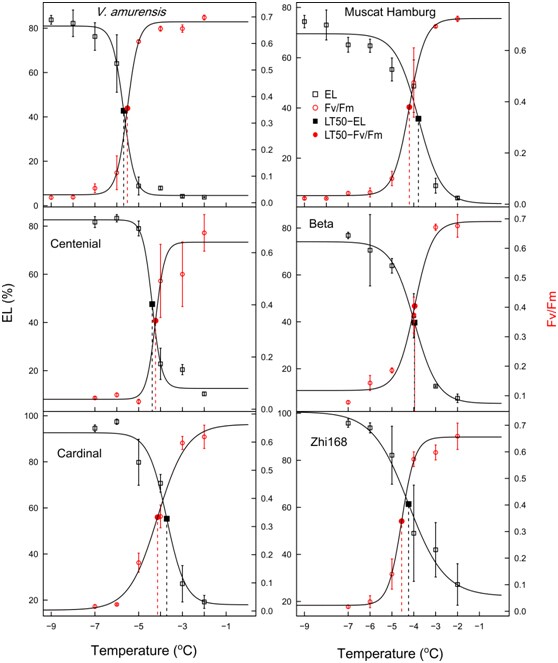

Supplement: Additional file 7: Figure S4. — Responses of electrolyte leakage and Fv/Fm as a function of temperature in different grape genotypes. The open symbols are observed mean ± SE with three replicates and lines are fitted curves to the Boltzmann 4−parameter model. Filled symbols indicate where the LT50 were estimated and the corresponding genotype is indicated in each figure. [file 12870_2015_459_MOESM7_ESM.jpeg]

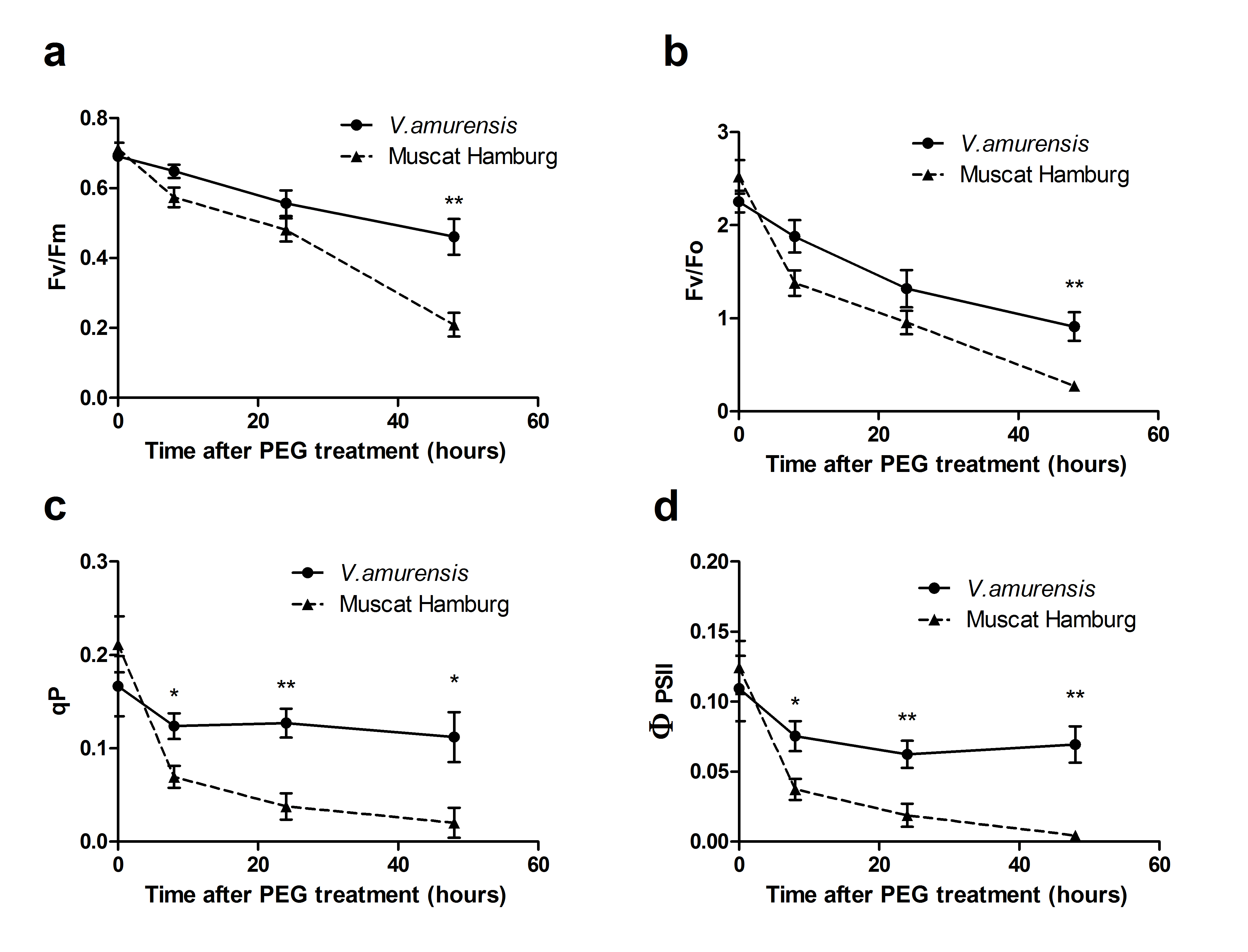

Supplement: Additional file 8: Figure S5. — Comparison of Fv/Fm (a), Fv/Fo (b), qP (c) and ϕPSII (d) at different time after PEG 6% treatment in V. amurensis and ‘Muscat Hamburg’. The values were the mean value ± SE of results from five replicates. * and ** indicate significant differences between V. amurensis and ‘Muscat Hamburg’ at P< 0.05 and P<0.01 level (t test), respectively. [file 12870_2015_459_MOESM8_ESM.jpeg]
