# Supplementary material for: A novel system for evaluating drought–cold tolerance of grapevines using chlorophyll fluorescence
Source: BMC Plant Biol. 2015 Mar 11;15:82. doi: 10.1186/s12870-015-0459-8 (PMC4367880; doi:10.1186/s12870-015-0459-8)
Supplement: Additional file 4: Table S1. — Summary statistics of linear regressions between the electrolyte leakage and the chlorophyll fluorescence parameter Fv/Fm under gradient cooling mode in six different grape genotypes. Slopes and intercepts are estimated by standard major axis regressions for each genotype. Their 95% confidence intervals (CI) are also provided. Different letters indicate significant differences in the intercept or slope among the genotypes at P< 0.05. [file 12870_2015_459_MOESM4_ESM.docx]

**Table S1** Summary statistics of linear regressions between the electrolyte leakage and the chlorophyll fluorescence parameter Fv/Fm under gradient cooling mode in six different grape genotypes. Slopes and intercepts are estimated by standard major axis regressions for each genotype. Their 95% confidence intervals (CI) are also provided. Different letters indicate significant differences in the intercept or slope among the genotypes at *P*< 0.05.

| Genotype | Intercept | |  | Slope | | *r*^2^ | *P*-value |
| --- | --- | --- | --- | --- | --- | --- | --- |
|  | Estimated | 95% CI |  | Estimated | 95% CI |  |  |
| *V. amurensis* | 82.8bc | 78.5 to 87.0 |  | -116.2a | -124.9 to -107.9 | 0.99 | < 0.0001 |
| Muscat Hamburg | 73.1c | 63.7 to 82.5 |  | -90.7a | -114.2 to -72.1 | 0.93 | < 0.0001 |
| Centenial | 85.3abc | 77.4 to 93.2 |  | -115.3a | -133.7 to -99.5 | 0.98 | < 0.0001 |
| Beta | 86.0b | 82.3 to 89.6 |  | -114.7a | -122.7 to -107.2 | 0.99 | < 0.0001 |
| Cardinal | 101.4a | 91.9 to 111.0 |  | -124.3a | -148.1 to -104.3 | 0.98 | < 0.0001 |
| Zhi168 | 98.4ab | 88.9 to 107.8 |  | -102.4a | -123.7 to -84.7 | 0.97 | <0.0001 |
