# Supplementary material for: A novel system for evaluating drought–cold tolerance of grapevines using chlorophyll fluorescence
Source: BMC Plant Biol. 2015 Mar 11;15:82. doi: 10.1186/s12870-015-0459-8 (PMC4367880; doi:10.1186/s12870-015-0459-8)
Supplement: Additional file 5: Table S2. — Correlation between electrolyte leakage (EL) and four chlorophyll fluorescence parameters (Fo, Fm, Fv/Fm and Fv/Fo) under gradient cooling mode in six different grape genotypes. [file 12870_2015_459_MOESM5_ESM.docx]

**Table S2 Correlation between electrolyte leakage (EL) and four chlorophyll fluorescence parameters (Fo, Fm, Fv/Fm and Fv/Fo) under gradient cooling mode in six different grape genotypes.**

| Genotype | EL− *Fo* | EL− *Fm* | EL− *Fv/Fm* | EL− *Fv/Fo* |
| --- | --- | --- | --- | --- |
| *V. amurensis* | 0.79 | 0.62 | 0.99 | 0.95 |
| Muscat Hamburg | 0.82 | 0.16 | 0.94 | 0.96 |
| Centenial | 0.21 | 0.78 | 0.98 | 0.81 |
| Beta | 0.76 | 0.84 | 0.99 | 0.97 |
| Cardinal | 0.8 | 0.77 | 0.98 | 0.98 |
| Zhi168 | 0.97 | 0.64 | 0.97 | 0.99 |
| Total | 0.57 | 0.57 | 0.9 | 0.88 |
