# Supplementary material for: A novel system for evaluating drought–cold tolerance of grapevines using chlorophyll fluorescence
Source: BMC Plant Biol. 2015 Mar 11;15:82. doi: 10.1186/s12870-015-0459-8 (PMC4367880; doi:10.1186/s12870-015-0459-8)
Supplement: Additional file 6: Table S3. — Significant analysis of LT50−EL and LT50−Fv/Fm data in V. amurensis and ‘Muscat Hamburg’ under two different cooling modes. * indicates significant differences between LT50−EL and LT50−Fv/Fm at P<0.05 level (paired t test). [file 12870_2015_459_MOESM6_ESM.docx]

**Table S3** Significant analysis of LT50−EL and LT50−Fv/Fm data in *V. amurensis* and ‘Muscat Hamburg’ under two different cooling modes. * indicates significant differences between LT50−EL and LT50−*Fv/Fm* at *P*< 0.05 level (paired t test).

| Mode | P value | Significant analysis |
| --- | --- | --- |
| Gradient cooling | 0.294 |  |
| Non-acclimated freezing | 0.012 | * |
